# Comparison of azithromycin plus chloroquine *versus* artemether-lumefantrine for the treatment of uncomplicated *Plasmodium falciparum* malaria in children in Africa: a randomized, open-label study

**DOI:** 10.1186/s12936-015-0620-8

**Published:** 2015-03-10

**Authors:** Richa Chandra, Patrick Ansah, Issaka Sagara, Ali Sie, Alfred B Tiono, Abdoulaye A Djimde, Qinying Zhao, Jeffery Robbins, Louis K Penali, Bernhards Ogutu

**Affiliations:** Novartis, Cambridge, MA USA; Navrongo Health Research Centre, Ghana Health Service War Memorial Hospital, Navrongo, Ghana; Malaria Research and Training Center, University of Sciences, Techniques and Technologies, Bamako, Mali; Centre de Recherche en Santé de Nouna, Nouna, Burkina Faso; Centre National de Recherche et de Formation sur le Paludisme, Ouagadougou, Burkina Faso; Global Research and Development, Pfizer Inc, 445 Eastern Point Road, Groton, CT 06340 USA; Institut Pasteur de Côte d’ Ivoire, Unité de Paludologie, Abidjan, Côte d’Ivoire; Walter Reed Project, Centre for Clinical Research, Kenya Medical Research Institute, Kisumu, Kenya

**Keywords:** Uncomplicated malaria, Azithromycin, Chloroquine, Paediatrics, Africa, Artemether-lumefantrine, Azithromycin-chloroquine, Treatment efficacy

## Abstract

**Background:**

This randomized, open-label study was conducted to establish the non-inferiority of a combination of azithromycin (AZ) and chloroquine (CQ) to artemether-lumefantrine (AL) for treatment of uncomplicated malaria in children from six sites in sub-Saharan Africa.

**Methods:**

Children with uncomplicated *Plasmodium falciparum* malaria between six and 59 months of age were randomized 1:1 to either AZCQ (30 mg/kg AZ + 10 mg/kg CQ base) or AL per prescribing information for three days (Days 0, 1, 2). Each site could enrol in the study population once the treatment of uncomplicated malaria in five children five to 12 years of age was deemed to be effective and well tolerated. The primary efficacy evaluation was the proportion of subjects in both the modified intent-to-treat (MITT) and per-protocol (PP) populations with an adequate clinical and parasitological response (PCR corrected) at Day 28. Non-inferiority was concluded if the lower bound of the 95% confidence interval comparing the two groups was 10 percentage points or greater.

**Results:**

A total of 255 children were enrolled in the efficacy analysis (AZCQ, n = 124; AL, n = 131). The PCR corrected clearance rates were 89% (AZCQ) *versus* 98% (AL) for MITT, a difference of -9.10 (95% confidence interval; -16.02, -2.18) and 93% (AZCQ) *versus* 99% (AL) for PP, a difference of -6.08 (-12.10, -0.05). Early and late treatment failures were more common in subjects receiving AZCQ. Adverse events were more common in subjects treated with AZCQ. Drug concentrations obtained at specified time points following AZCQ administration had a large coefficient of variation partially due to sparse sampling with sample collection time window.

**Conclusions:**

In this study, non-inferiority of AZCQ to AL was not demonstrated.

**Trial registration:**

ClinicalTrials.gov NCT00677833.

**Electronic supplementary material:**

The online version of this article (doi:10.1186/s12936-015-0620-8) contains supplementary material, which is available to authorized users.

## Background

Malaria, a disease caused by five different species of *Plasmodium*, continues to be a global health problem [1]. Throughout the world, approximately 207 million cases of malaria were reported in 2012, 80% in Africa. *Plasmodium falciparum* accounted for 91% of total cases. The vast majority of the estimated 627,000 malaria deaths occurred in 2012, 90% in Africa, and children under five years accounted for 77% of these deaths [1]. The World Health Organization (WHO) recommends an artemisinin-based combination therapy (ACT) for uncomplicated malaria caused by *P. falciparum* [2]*.*

The combination of azithromycin (AZ) and chloroquine (CQ) has been shown to have synergistic activity *in vitro* and *in vivo* against CQ-resistant strains of *P. falciparum* [3-5]. AZ and CQ are marketed drugs, and there is extensive experience with each of these agents in the paediatric population. The efficacy and safety of a fixed-dose combination of AZ and CQ (AZCQ) for the treatment of symptomatic, uncomplicated *P. falciparum* malaria in adults were recently demonstrated in two multicentre phase 3 clinical studies in Africa [6,7] and in phase 2 studies in India and Colombia [8-10]. In a separate study, a fixed-dose combination of AZCQ is currently undergoing evaluation for intermittent preventative treatment in pregnancy (IPTp) [11]. The objective of this study (ClinicalTrials.gov identifier NCT00677833) was to establish the non-inferiority of AZCQ compared with the ACT combination artemether-lumefantrine (AL) for treatment of symptomatic, uncomplicated *P. falciparum* malaria in African children.

## Methods

### Study design

This was an open-label, randomized, phase 2/3 study at six sites (two centres in Burkina Faso and one centre each in Kenya, Ghana, Mali, and Ivory Coast) in sub-Saharan Africa that compared the efficacy (adequate clinical and parasitological response (ACPR)) of AZCQ with that of AL in treatment of children with symptomatic uncomplicated falciparum malaria. An independent external data monitoring committee (EDMC), comprising international and regional malaria researchers, had oversight of the study. The study was conducted between June 2008 and September 2010.

### Subjects

To be eligible for participation in the study, subjects were required to be able to receive treatment on an outpatient basis. Subjects were included if age ranged from five to 12 years of age (screening cohort) or between six and 59 months of age (primary study cohort) and had uncomplicated, symptomatic malaria. The presence of malaria was defined as positive blood smears for *P. falciparum*, mono-infection with parasite counts between 1,000 to 100,000 parasites/μL, and documented fever (≥38.0°C, rectal or tympanic; or ≥37.2°C axillary or ≥37.5°C oral), or history of fever within the previous 24 hours. Subjects also had to have a blood glucose ≥60 mg/dL and haemoglobin ≥6 g/dL or haematocrit ≥18% without signs of anaemia-induced congestive heart failure, and a negative urine pregnancy test for females ≥ ten years of age. Informed consent for the study and permission from subjects’ legal guardian for the three-day inpatient stay was also needed. Concomitant use of medications (e.g., anti-emetics, antipyretics) was permitted. Use of medications with known drug interactions with macrolides was closely monitored. Medications metabolized by the CYP2D6 isoenzyme were not permitted (contra-indicated with AL). In addition, agents that prolong the QT interval and concomitant administration of other anti-malarial drugs were avoided because of limited safety data with AL.

Exclusion criteria included subjects with severe or complicated malaria or a peripheral blood smear indicating a mix of *Plasmodium* species, any history of allergy/hypersensitivity to or contraindication for use of any of the study drugs or history of treatment with anti-malarial drugs within two weeks prior to enrollment, body weight <5 kg or severe malnutrition, and known or suspected cardiovascular, hepatic, or renal abnormality, specific systemic diseases, or other medical conditions that would interfere with the evaluation of therapeutic response or safety of the study drug, other severe acute or chronic medical or psychiatric condition or laboratory abnormality, or other common febrile conditions, such as tonsillitis, measles, etc.

### Study procedure

In Burkina Faso, the National Ethical Committee for Health Research reviewed and approved the final protocol, informed consent, and any amendments. At all other study centres, this review and approval was done by the institutional review board at each study centre. Oversight of safety data evaluation was provided through the activities of the EDMC. This study was conducted in compliance with the ethical principles originating in or derived from the Declaration of Helsinki and in compliance with all International Conference on Harmonization Good Clinical Practice Guidelines. The study protocol was submitted to the US Food and Drug Administration under an Investigational New Drug application. All local regulatory requirements were also followed.

Subject enrolment was carried out in two stages. Each site was required to initially enrol ten evaluable subjects in a screening cohort (Cohort 1) of older children (five to 12 years of age) who were assumed to have some degree of immunity to *P. falciparum* before proceeding with the primary study population cohort (Cohort 2) of younger children (six to 59 months of age). The decision to proceed to Cohort 2 for each site was based on the safety and efficacy of five evaluable AZCQ subjects observed in Cohort 1 at that site. The site could begin enrolling in Cohort 2 if all five evaluable subjects in the AZCQ group of Cohort 1 demonstrated a clinical and parasitological response within the first seven days of treatment initiation, continued clearance of parasitaemia through Day 28, and no clinically significant safety and tolerability issues were noted. If four of five subjects in the AZCQ arm demonstrated parasitological clearance and a clinical response within the first seven days of treatment initiation, the centre continued to enrol approximately ten additional subjects with five evaluable subjects receiving AZCQ. Continuing onto Cohort 2 required that eight or more of ten AZCQ-treated subjects demonstrated a clinical response and parasitologic clearance up to Day 28, and no significant safety and tolerability issues were noted. If fewer than four of five subjects in the AZCQ arm met the above criteria, the EDMC evaluated data from all sites and made recommendations on when to proceed with Cohort 2 enrolment. In all other cases, the decision to move from Cohort 1 to Cohort 2 for each centre was made by the investigator, the sponsor (Pfizer), and occasionally the EDMC.

In each cohort, subjects were randomized 1:1 to either receive an AZCQ fixed-dose combination tablet (30 mg/kg AZ + 10 mg /kg CQ base) orally once daily for three days (Days 0, 1, 2) or AL orally per prescribing information for three days (Days 0, 1, 2). A computer-generated randomization list was provided to investigators and randomization numbers were assigned sequentially as subjects were deemed eligible to participate in the study. Random assignment to either the AZCQ or AL treatment groups was determined by the assigned number from the randomization list. Two strengths of AZCQ were available for this study: AZ 300 mg/CQ 100 mg and AZ 150 mg/CQ 50 mg. Tablets were scored to allow for dosing by body weight. Subjects received AZCQ 300/100-mg tablets for ≥20 kg body weight: and AZCQ 150/50-mg tablets for 5 to <20 kg body weight. When possible, the study drugs were administered with or immediately after food consumption. Any dose of AZCQ that was vomited within 30 minutes of administration and any dose of AL that was vomited within 60 minutes of administration was repeated once. If vomiting recurred, the subject was discontinued from the study treatment.

At enrolment, the presence of *P. falciparum* was identified using a blood dipstick-based test (Binax NOW immunochromatographic test (ICT)) and confirmed by microscopy on a Giemsa-stained peripheral blood smear. All eligible subjects were then hospitalized for ≥ three days for study drug administration and monitoring of the study key parameters. During this time, blood smears (thick and thin) were obtained every eight hours until two consecutive smears showed absence of parasitaemia. Clinical assessments, including adverse events (AEs), thick and thin peripheral smears and vital signs were evaluated on study Days 0, 3, 7, 14, 21, 28, 35, and 42. Electrocardiograms were performed in a limited number of subjects on study Days 0 and 2. At each site, clinical presentation and smear results from each study day were used to determine subject management. Discharge from the hospital occurred following clearance of asexual parasitaemia and investigator approval. All subjects were given insecticide-treated bed nets for use in the hospital and for use at home once discharged.

Sparse pharmacokinetic (PK) blood samples were collected only in the AZCQ treatment group to determine serum AZ, plasma CQ, and desethyl-CQ concentrations. Whole blood samples were collected on Day 0 (prior to the first dose) at 0 hours (window: -1 to 0 hour); on Day 2 (the day of the third dose) at 0 (pre-dose, window: -1 to 0 hr), three (window: 2–4 hours), and eight hours (window: 6–10 hours), and randomly on Day 7. Samples were either centrifuged at 3,500 rpm for 10 min at 4°C or the centrifuge bucket and rotor were chilled on ice or in a refrigerator for two hours prior to use. Samples were stored within one hour of collection at -20°C until shipped for assay. AZ, CQ and desethyl-CQ concentrations were analysed using validated, sensitive and specific high-performance liquid chromatography tandem mass spectrometric methods in compliance with Pfizer standard operating procedures. AZ assays were performed by Bioanalytical Systems, Ltd (BASi) (Warwickshire, UK) and CQ/desethyl-CQ assays were performed by Cetero Research (Houston, TX, USA) [12].

A prospectively planned interim analysis was conducted to evaluate safety and efficacy endpoints when approximately 50% of the subjects enrolled in Cohort 2 (approximately 52 evaluable subjects per arm) completed the Day 28 visit. Interim data were reviewed by the EDMC, and a determination to continue the study was made based on a predefined futility criterion (ACPR). ACPR was defined as asexual *P. falciparum* parasitologic clearance at Day 28 irrespective of axillary, oral, rectal, or tympanic temperature without previously meeting the criteria of early treatment failure or polymerase chain reaction (PCR)-corrected late treatment failure. *PCR-corrected* refers to the use of molecular testing to differentiate recrudescence from re-infection when evaluating efficacy (see Table 1 for full definitions). Filter-paper samples were used for molecular analyses and gene amplification of merozoite surface proteins (MSP-1, MSP-2), Ca1, TA-87, and TA-99 polymorphisms to distinguish recrudescence from new infections [13-16]. A subject was classified as a true failure if the sample was recrudescent with all five genotypic markers (i.e., all five markers matched those found at baseline screening). Cases of post-treatment mixed infection that contained parasites genotypically identical to those from Day 0 were counted as recrudescent. Change of profile in any or all loci was counted as re-infection. Although the study was open-label, the study team was blinded to the emerging cumulative data reviewed by the independent external EDMC.

**Table 1:** **Treatment failure definitions** [17]

**Early treatment failure (ETF)**	● Signs of severe malaria/clinical deterioration requiring rescue medication on Days 0, 1, 2 or 3, in the presence of *P. falciparum* parasitaemia*
	● Last available asexual *P. falciparum* parasite count on Day 2 greater than the first available parasite count at baseline irrespective of axillary, oral, or rectal temperature
	● Parasitaemia (*P. falciparum*) on Day 3 with fever^†^
	● Last available *P. falciparum* parasite count on Day 3 ≥ 25% of the first available parasite count at baseline
**Late treatment failure**	
Late clinical failure (LCF)	● Signs of severe malaria/ clinical deterioration requiring rescue medication after Day 3 in the presence of *P. falciparum* parasitaemia, without previously meeting any of the criteria of ETF
	● Presence of *P. falciparum* parasitaemia and fever^†^ on any day from Day 4 onward, without previously meeting any of the criteria of ETF
Late parasitologic failure	● Presence of *P. falciparum* parasitaemia on any day from Day 7 onward and the absence of fever without previously meeting any of the criteria of ETF or LCF

*For treatment failure, any subject with a missing blood smear was assumed to have parasitaemia.

^†^Fever was defined as ≥38.0°C (rectal), ≥37.2°C (axillary), or ≥37.5°C (oral).

### Laboratory assessment

Peripheral blood smear examinations were conducted to evaluate eligibility for inclusion by assessing parasite counts and identifying parasite species, parasite susceptibility to CQ and response to treatment. Molecular testing was done to genotype the *P. falciparum* parasites at baseline and when parasitaemia occurred after initial clearance. Recurrence (reappearance of asexual *P. falciparum* parasitaemia following a quiescent or latent period after cessation of the primary attack) was categorized as either recrudescence (reappearance of asexual *P. falciparum* blood stage parasites of same genotype confirmed by molecular testing), or re-infection (infection by a different genotypic parasite as documented by molecular testing).

### Statistical analysis

Cohort 1 and Cohort 2 were analysed independently. Because Cohort 1 was only a small screening cohort, although data were analysed, no formal statistical inference was made. Three study populations were defined: 1) all treated subjects (for safety analyses); 2) modified intent-to-treat (MITT), defined as a subset of subjects in the all-treated subjects population who met the specified disease criteria at baseline; and, 3) the per-protocol (PP) population, defined as a subset of subjects in the MITT population who received all three days of assigned study medication.

External quality checks were conducted for on-site microscopists. At Ivory Coast, all three microscopists failed the parasite-count evaluation; therefore, data (nine subjects) from Ivory Coast were excluded from all ACPR analyses for both the MITT and PP populations, but included in sensitivity analysis for the MITT population.

The primary efficacy endpoint was the proportion of subjects with PCR-corrected ACPR at the Day 28 evaluation. Two datasets were analysed for efficacy: the MITT and PP populations (excluding data from Ivory Coast). Cohort 2 was the focus of the efficacy analysis. The primary analysis variable was the time (days) to the first occurrence of treatment failure analysed using the Kaplan-Meier method (see Table 1 for categorization of treatment failure events). Subjects without a treatment failure event during the study were censored using the Kaplan-Meier method as listed below.

- Subjects who withdrew from the study due to any reason other than treatment failure were censored on the day of the last available blood smear measurement.
- Subjects who received an anti-malarial concomitant medication for treatment of a reinfection (in a PCR-corrected analysis) were censored at the visit date if the anti-malarial was given after collection of the blood smear on that day. Otherwise, the subject was censored on the day of the last available blood smear measurement taken prior to the visit. Note that in a PCR-uncorrected analysis, this type of censoring only occurred when a subject was treated at a visit based on symptoms but later found not to have parasitaemia.

The proportion of subjects with ACPR at each planned visit (i.e., Days 7, 14, 21, 28 (primary), 35, 42) was estimated from the Kaplan-Meier curve. A two-sided 95% confidence interval (CI) for the difference in ACPR proportions (AZCQ – AL) was constructed. Non-inferiority was concluded if the lower boundary of the CI was greater than or equal to -10 percentage points for both the MITT and PP populations at Day 28 (PCR-corrected).

A sensitivity analysis was conducted to support the primary efficacy analysis for both the MITT and PP populations. This sensitivity analysis considered subjects who prematurely discontinued from the study due to any reason, without having an event, as treatment failures at the time of withdrawal.

Secondary study endpoints included ACPR (PCR-corrected and -uncorrected) at Days 7, 14, 21, 28 (uncorrected), 35, and 42 as well as early and late treatment failures (Table 1), time to fever clearance, and gametocyte clearance. The time to fever clearance was analysed in a similar manner to ACPR, with Kaplan-Meier estimates. Clearance was defined as the time (in days) from the start of treatment administration to the first of the two consecutive time points at which fever was no longer present. Absence of gametocytes was defined as clearance of *P. falciparum* gametocytaemia (i.e., attainment of two consecutive zero gametocyte counts) without subsequent recurrence through the day of consideration. With the exception of ACPR, all other secondary endpoints were analysed using the MITT study population. Descriptive statistics of concentration data for serum AZ, plasma CQ and plasma desethyl-CQ were provided by treatment, cohort, study day, and nominal time post-dose.

A target sample size of approximately 244 subjects in Cohort 2 (approximately 122 subjects per arm) was determined based on the following assumptions: 1) an evaluability rate (for the purpose of sample size determination) of 85% corresponding to the Day 28 visit (this would result in 104 evaluable subjects in each treatment arm); 2) an expected ACPR (PCR-corrected) rate of 95% in the AZCQ group and 95% in the AL group; 3) a power of 80% to show non-inferiority of the AZCQ group relative to the AL group; and, 4) accounting for any loss in power due to the efficacy futility interim analysis.

## Results

### Cohort 1

The screening cohort included 106 subjects. After excluding subjects from the Ivory Coast, the MITT population included 85 subjects. Four subjects or their parent/legal guardian in the AZCQ treatment group were no longer willing to participate and discontinued from the study. There were no treatment discontinuations in the AL group. The mean age was 7.4 years for AZCQ-treated subjects and 7.9 years for AL-treated subjects. The AL group had a higher proportion of males. All subjects had confirmed *P. falciparum* malaria following blood smear microscopy with mean baseline parasite counts that ranged from 1,000/μL to 110,240/μL. All study centres met the criteria for enrolling subjects into Cohort 2.

### Cohort 2

#### Subject disposition and demographics

**Figure 1:**
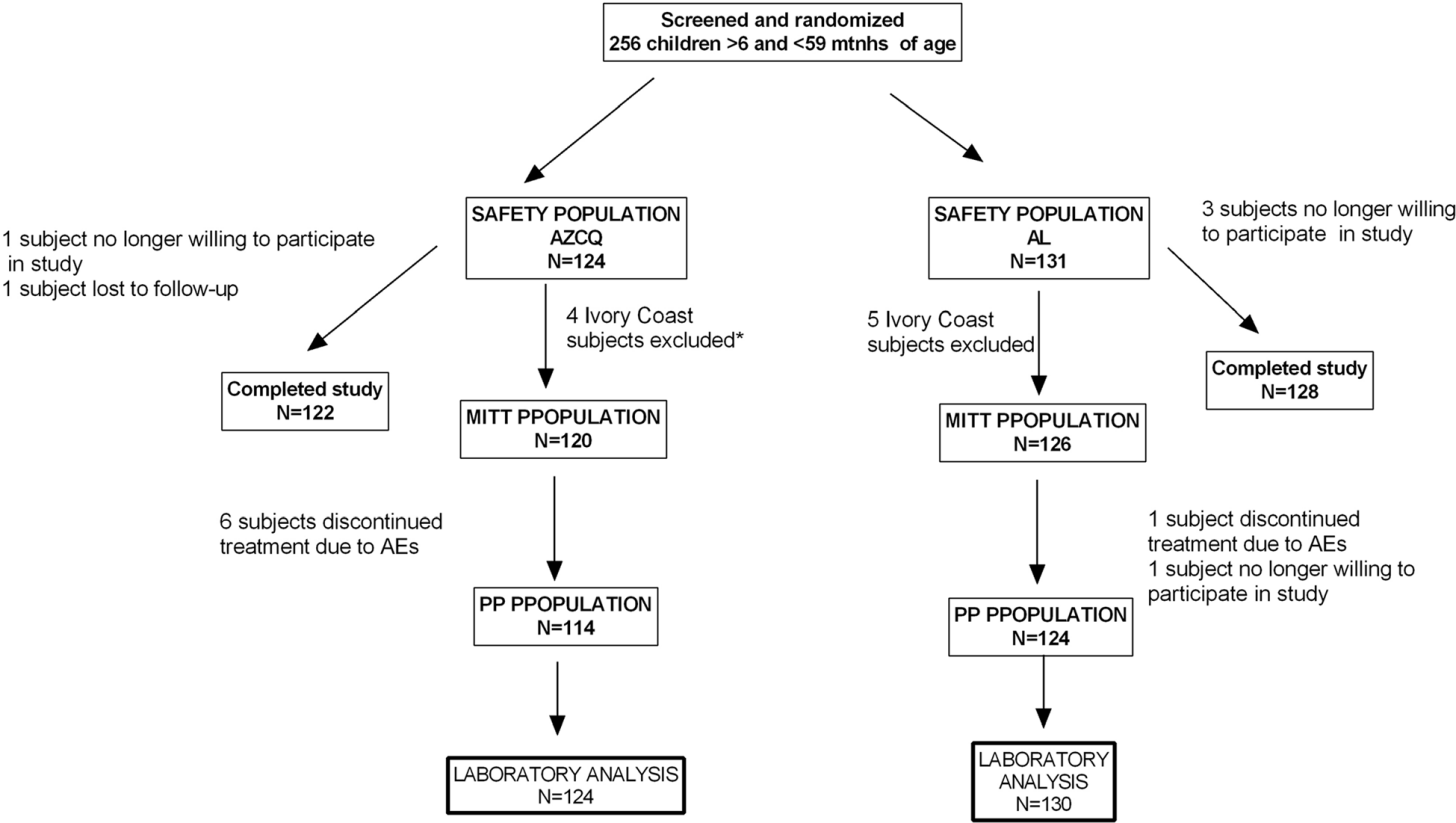
**Subject disposition (Cohort 2).** AE = adverse event, AL = artemether-lumefantrine, AZCQ = azithromycin-chloroquine fixed-dose combination, MITT = modified intent-to-treat, PP = per protocol. *Includes 1 subject who discontinued treatment due to an AE.

The primary study population included 255 subjects who were screened and randomized (Figure 1). After excluding subjects from the Ivory Coast, the MITT population included 246 subjects. One subject or their parent/legal guardian in the AZCQ group was no longer willing to participate and discontinued from the study, and one subject was lost to follow-up. Treatment discontinuation due to AEs occurred in seven subjects in the AZCQ group. Three subjects or their parent/legal guardian in the AL treatment group were no longer willing to participate and discontinued from the study, and one subject stopped treatment because of AEs.

Demographic characteristics of treatment groups were similar, except for gender ratio; there was a higher proportion of males in the AZCQ group (Table 2). The mean age was 2.4 years for the AZCQ group and 2.7 years for the AL group.

**Table 2:** **Demographic characteristics (Cohort 2)**

	**AZCQ**	**AL**
	**N = 124**	**N = 131**
Male (n, %)	74 (59.7)	66 (50.4)
6 months to <5 years (n, %)	123 (99.2)	129 (98.5)
5 years (n, %)*	1 (0.8)	2 (1.5)
Age (years) mean (SD)	2.4 (1.3)	2.7 (1.0)
Range	0.5-5.0	0.5-5.0
Weight (kg) mean (SD)	12.5 (3.1)	12.8 (2.5)
Range	6.1-19.0	6.9-18.0
Height (cm), mean (SD)	90.2 (11.6)	92.7 (9.5)
Range	59.0-115.0	66.3-116.0
Body mass index (kg/m^2^) mean (SD)	15.2 (1.6)	14.9 (1.6)
Range	9.5-20.7	8.4-20.3

AL = artemether-lumefantrine, AZCQ = azithromycin-chloroquine fixed-dose combination, SD = standard deviation.

*Three subjects who were 5 years of age were protocol violations.

#### Baseline disease characteristics

At baseline, all subjects had either fever or a history of fever within 24 hours of consent to participate in the study. The mean duration between subjects’ first malaria symptom and initiation of consent process for this study was 1.9 days, and ≤ three hours elapsed between consent and drug administration in all but 12 subjects.

#### Efficacy results

Efficacy data were used to determine if AZCQ was non-inferior to AL; based on the primary endpoint of ACPR at Day 28 (PCR-corrected), non-inferiority of AZCQ to AL was not demonstrated. In the MITT population, 89% (95% CI: 83%, 96%) of subjects in the AZCQ group *versus* 98% (95% CI: 96%, 100%) in the AL group achieved the primary endpoint (Table 3). The difference between treatment groups in the MITT (AZCQ-AL) was -9% (95% CI, -16%, -2%), which did not meet the pre-specified non-inferiority criterion (lower bound of the CI ≥ -10% was needed to conclude non-inferiority). Results for the PP population were similar to the MITT. In the PP population, 93% (95% CI: 87%, 99%) in the AZCQ group *versus* 99% (95% CI: 97%, 100%) in the AL group achieved the primary endpoint (Table 3)**.** The between-group differences in the PP population (AZCQ-AL) was -6% (95% CI, -12%, -0.1%), which also did not meet the non-inferiority criterion. Although not a pre-specified comparison in the study protocol, since the CI for both the MITT and PP populations also excluded zero, AZCQ was inferior to AL at the Day 28 time point.

**Table 3:** **ACPR (PCR-corrected) at Days 7, 14, 21, 28, 35, 42 (Cohort 2)**

**Percentage of subjects with ACPR** ^*^ **(95% CI)** ^**†**^	**MITT population**		**PP population**	
	**AZCQ N = 120**	**AL N = 126**	**AZCQ N = 114**	**AL N = 124**
Day 7	94.17 (89.55, 98.78)	99.21 (97.25, 100.00)	98.25 (95.39, 100.00)	100.00 (−)
ACPR % difference^‡^, 95% CI^†^	-5.04 (-9.93, -0.15)		-1.75 (−)	
Day 14	92.47 (87.30, 97.64)	99.21 (97.24, 100.00)	96.46 (92.59, 100.00)	100.00 (−)
ACPR % difference^‡^, 95% CI^†^	-6.74 (-12.15, -1.32)		-3.54 (−)	
Day 21	91.59 (86.04, 97.14)	98.37 (95.65, 100.00)	95.53 (91.10, 99.96)	99.16 (97.03, 100.00)
ACPR % difference^‡^, 95% CI^†^	-6.78 (-12.82, -0.75)		-3.63 (-8.40, 1.14)	
Day 28^§^	89.27 (82.77, 95.77)	98.37 (95.59, 100.00)	93.08 (87.32, 98.84)	99.16 (96.97, 100.00)
ACPR % difference^‡^, 95% CI^†^	-9.10 (-16.02, -2.18)		-6.08 (-12.10, -0.05)	
Day 35	89.27 (82.68, 95.86)	96.19 (91.85, 100.00)	93.08 (87.22, 98.95)	96.96 (92.90, 100.00)
ACPR % difference^‡^, 95% CI^†^	-6.92 (-14.59, 0.76)		-3.87 (-10.79, 3.04)	
Day 42	87.55 (80.08, 95.03)	96.19 (91.79, 100.00)	91.29 (84.31, 98.28)	96.96 (92.84, 100.00)
ACPR % difference^‡^, 95% CI^†^	-8.63 (-17.08, -0.18)		-5.66 (-13.55, 2.22)	

ACPR = adequate clinical and parasitologic response, AL = artemether-lumefantrine, AZCQ = azithromycin-chloroquine fixed-dose combination, CI = confidence interval, MITT = modified intention-to-treat, PP = per protocol.

^*^Estimated from Kaplan-Meier curve.

^†^CI by large sample approximation to the binomial with continuity correction using the standard error estimated by the Greenwood formula.

^‡^Difference calculated from rates estimated from the Kaplan-Meier curves.

^§^Primary endpoint.

Note: Group-specific confidence intervals and the confidence interval for the difference between treatment groups were not calculated when all failures occurred in a single group.

The proportion of subjects with ACPR (PCR-corrected) was numerically higher in the AL group than in the AZCQ group at all visits for both analysis populations (Table 3). A significant difference between groups was noted at all visits except Day 35 in the MITT population. The PCR-uncorrected results (Table 4) were markedly lower than those that were PCR-corrected starting at Day 21 onward. In the MITT population, proportion of PCR-uncorrected ACPR was 52% of subjects in the AZCQ group *versus* 73% in the AL group at Day 28, with similar findings in the PP population.

**Table 4:** **ACPR (PCR-uncorrected) at Days 7, 14, 21, 28, 35, 42 (Cohort 2)**

**Percentage of subjects with ACPR** ^*^ **(95% CI)** ^**†**^	**MITT population**		**PP population**	
	**AZCQ N = 120**	**AL N = 126**	**AZCQ N = 114**	**AL N = 124**
Day 7	94.17 (89.55, 98.78)	99.21 (97.25, 100.00)	98.25 (95.39, 100.00)	100.00 (−,-)
ACPR % difference^‡^, 95% CI^†^	-5.04 (-9.93, -0.15)		-1.75 (−.-)	
Day 14	89.08 (83.05, 95.11)	96.79 (93.28, 100.00)	92.89 (87.69, 98.08)	97.56 (94.42, 100.00)
ACPR % difference^‡^, 95% CI^†^	-7.71 (-14.54, -0.88)		-4.67 (-10.6, 1.25)	
Day 21	67.87 (59.02, 76.72)	82.96 (75.91, 90.01)	70.56 (61.67, 79.45)	83.62 (76.65, 90.60)
ACPR % difference^‡^, 95% CI^†^	-15.09 (-26.24, -3.94)		-13.07 (-24.21, -1.92)	
Day 28	51.55 (42.07, 61.02)	73.31 (65.10, 81.52)	54.28 (44.57, 63.98)	73.90 (65.71, 82.08)
ACPR % difference^‡^, 95% CI^†^	-21.79 (-34.14, -9.39)		-19.62 (-32.16, -7.08)	
Day 35	44.67 (35.24, 54.11)	62.91 (54.00, 71.82)	47.04 (37.31, 56.77)	63.41 (54.49, 72.34)
ACPR % difference^‡^, 95% CI^†^	-18.24 (-31.05, -5.43)		-16.38 (-29.42, -3.33)	
Day 42	37.80 (28.58, 47.02)	56.29 (47.12, 65.46)	39.80 (30.24, 49.36)	56.74 (47.54, 65.94)
ACPR % difference^‡^, 95% CI^†^	-18.49 (-31.33, -5.65)		-16.94 (-30.04, -3.83)	

ACPR = adequate clinical and parasitologic response, AL = artemether-lumefantrine, AZCQ = azithromycin-chloroquine fixed-dose combination, CI = confidence interval, MITT = modified intention-to-treat, PP = per-protocol.

^*^Estimated from Kaplan-Meier curve.

^†^CI by large sample approximation to the binomial with continuity correction using the standard error estimated by the Greenwood formula.

^‡^Difference calculated from rates estimated from the Kaplan-Meier curves.

Note: Group-specific confidence intervals and the confidence interval for the difference between treatment groups was not calculated when all failures occurred in single group.

Most (eight of 12 and one of two in the AZCQ and AL groups, respectively) of the treatment failures (up to Day 28 including early treatment failures) were from a single centre (Mali). Estimates of ACPR at Day 28 (MITT) were essentially unchanged when accounting for each subject’s baseline parasite count (i.e., treatment groups compared within two subgroups based on whether a subject’s baseline parasite count was above or below the median observed value of 13,120). Early treatment failures were more common in the AZCQ group (5.83% (MITT) and 1.75% (PP)) than in the AL group (0.79% (MITT) and 0% (PP)). Also, higher proportions of late parasitological failures were observed in the AZCQ group (4.17% (MITT) and 4.39% (PP)) than in the AL group (0.79% (MITT) and 0.81% (PP)). No late clinical failures were observed in either treatment group (MITT or PP).

The time to fever clearance between groups was approximately 24 hours in both treatment groups with no statistically significant difference between groups. One subject in each treatment group had fever that persisted for more than 72 hours despite being aparasitaemic from study Day 1 (AL) or study Day 2 (AZCQ). *Plasmodium falciparum* gametocyte clearance rates were higher at all time points for AL-treated subjects (>90%) compared with that for AZCQ-treated subjects (>80%).

#### Pharmacokinetic results

Serum concentrations of AZ and plasma concentrations of CQ and desethyl-CQ in each cohort and for the combination of cohorts are reported in Table 5. A large coefficient of variation, ranging from 39 to 93%, was observed in the concentration data and may have been caused partially by the PK sampling time window and approximate weight-based dose according to design.

**Table 5:** **Descriptive summary of serum azithromycin and plasma chloroquine/desethylchloroquine concentrations**

**Study day**	**Time post dose (hrs)**			
		Mean (% CV) azithromycin serum concentrations*		
		**Cohort 1**	**Cohort 2**	**Cohorts 1 and 2 combined**
0	0	0 N = 52	0 N = 119	0 N = 171
2	0	216 (39) N = 49	195 (93) N = 114	201 (78) N = 163
2	3	1160 (44) N = 49	906 (58) N = 111	938 (54) N = 160
2	8	609 (48) N = 49	467 (59) N = 115	510 (56) N = 166
7	NS	44.7 (67) N = 48	26.9 (75) N = 118	320 (77) N = 166
		Mean (% CV) chloroquine plasma concentrations*		
0	0	0 N = 44	0 N = 107	0 N = 151
2	0	172 (35) N = 49	131 (53) N = 110	144 (48) N = 159
2	3	430 (36) N = 48	331 (44) N = 107	362 (43) N = 155
2	8	367 (43) N = 49	295 (40) N = 106	318 (43) N = 155
7	NS	54.5 (67) N = 49	35.1 (53) N = 112	41.0 (65) N = 166
		Mean (% CV) desethylchloroquine plasma concentrations*		
0	0	0 N = 43	0 N = 103	0 N = 146
2	0	95.1 (77) N = 49	77.5 (67) N = 110	82.9 (72) N = 159
2	3	170 (48) N = 49	138 (48) N = 107	148 (49) N = 156
2	8	160 (58) N = 49	147 (58) N = 104	151 (58) N = 153
7	NS	66.9 (94) N = 49	38.5 (87) N = 112	46.8 (97) N = 161

*Concentration is in ng/mL.

CV = coefficient of variation, NS = not specified.

#### Safety results

The safety population included 361 subjects randomized in this study. No deaths occurred during the study. Four serious AEs (SAEs) were reported. Three SAEs occurred in Cohort 1 (malaria (AZCQ group), sepsis and hepatitis B (one each, AL group)), and one SAE (convulsions, AL group) in Cohort 2. None of these SAEs were considered related to study drug by investigators. Seven Cohort 2 subjects (5.6%) in the AZCQ group withdrew from treatment due to an AE, *versus* one (0.8%) in the AL group. No meaningful differences in laboratory parameters were noted between AZCQ and AL treatment groups (See Additional file 1).

The most frequently reported AEs in Cohort 2 overall were asymptomatic parasitaemia (coded as infection parasitic), vomiting, malaria, and pyrexia (Table 6). The most frequently reported AEs in the AZCQ group were vomiting (30.6%), asymptomatic parasitaemia (29.8%) and malaria (21.0%). Those AEs most frequently reported and considered related to AZCQ treatment were vomiting (27.4%), asymptomatic parasitaemia (10.5%) and pruritus (6.5%). Vomiting was a common reason for discontinuation from dosing in the AZCQ group. Vomiting with AZCQ generally resolved quickly (within the day of onset) and appeared to be related to the AZCQ drug combination rather than an age-specific tolerability issue because it was observed at a higher frequency in both cohorts. The most frequently reported AEs in the AL group were asymptomatic parasitaemia (23.7%), pyrexia (20.6%) and malaria (14.5%), with pyrexia (10.7%), asymptomatic parasitaemia (10.7%) and vomiting (7.6%) considered related to treatment. Most AEs across both treatment groups were mild to moderate in severity.

**Table 6:** **Treatment-emergent adverse events occurring in ≥5% of subjects in Cohort 2 (all causalities)**

**System organ class preferred term, n (%)**	**AZCQ N = 124**	**AL N = 131**
Gastrointestinal disorders		
Vomiting*	38 (30.6)	13 (9.9)
Abdominal pain^†^	4 (3.2)	14 (10.7)
Diarrhoea	4 (3.2)	8 (6.1)
Infections and infestations		
Infection parasitic	37 (29.8)	31 (23.7)
Malaria	26 (21.0)	19 (14.5)
Upper respiratory tract infection	9 (7.3)	12 (9.2)
Bronchitis	4 (3.2)	9 (6.9)
Respiratory tract infection	2 (1.6)	8 (6.1)
General disorders and administration site conditions		
Pyrexia	17 (13.7)	27 (20.6)
Respiratory, thoracic and mediastinal disorders		
Cough	15 (12.1)	13 (9.9)
Metabolism and nutrition disorders		
Decreased appetite	9 (7.3)	5 (3.8)
Nervous system disorders		
Skin and subcutaneous tissue disorders		
Pruritus	8 (6.5)	2 (1.5)

Medical Dictionary for Regulatory Activities (version 13.1) coding dictionary applied.

*p < 0.0001 and ^†^p < 0.05 using Fisher’s exact test.

## Discussion

Based on the predefined primary endpoint in this study, non-inferiority of AZCQ relative to AL was not demonstrated. Although both treatment groups had a high proportion of subjects achieving ACPR at Day 28 (PCR-corrected), the proportion was lower (i.e. inferior) in the AZCQ group compared with the AL group.

In the PP population, the efficacy was 93% in AZCQ-treated subjects compared with 99% in the AL-treated subjects at Day 28. Similarly, in the MITT, efficacy was 89% in AZCQ-treated subjects compared with 98% in AL-treated subjects. The conclusions from the sensitivity analyses performed for all ACPR endpoints, including subjects from the Ivory Coast and classifying study discontinuations as failures, were no different from the main study conclusion. The evaluability criteria may explain the small difference in AZCQ efficacy observed between the PP and MITT populations, since subjects in the PP population were required to receive all three days of study medication. This study used the Kaplan-Meier curve/product limit estimator to evaluate the primary endpoint. Use of this limit estimator is recommended by WHO [18] and has been used in analysing the primary efficacy endpoint in late-stage anti-malarial treatment clinical trials [19,20].

Absence of non-inferiority of AZCQ observed in this study was an unexpected outcome because there is evidence of effectiveness of AZ in combination with CQ and other agents for treatment of malaria. In two randomized, comparative studies in African adults with *P. falciparum* malaria, treatment with AZ 1,000 mg and CQ 600 mg resulted in Day 28 PCR-corrected parasitologic clearance rates of 98% [21]. In one study, AZ in combination with artesunate was compared with AL in a population that included children (≥eight years of age) and adults with uncomplicated *P. falciparum* malaria in Bangladesh [22]. Although the 42-day cure rate was numerically lower (94.6%) for AZ-artesunate than for AL (97%), the difference was not statistically significant and at a level that was therapeutically beneficial. This study was not powered for equivalence. In another study, AZ was combined with artesunate and compared with AL for treatment of uncomplicated *P. falciparum* malaria in children six to 59 months of age in Tanzania [23]. Treatment failures were higher in the AZ-artesunate group, however, the dose of AZ used was lower than in the current study (20 mg/kg *versus* 30 mg/kg). In addition, CQ, alone and in combination with artesunate, AZ, or atovaquone-proguanil, has been used to treat uncomplicated *P. falciparum* malaria in similar-aged children in Malawi [24]. In this study, there were no identified strains of CQ-resistant *P. falciparum,* and the incidence of malaria was similar across the treatment groups.

In the present study, the serum AZ and plasma CQ concentrations were generally comparable between Cohorts 1 and 2. The large coefficient of variation may limit the ability to draw conclusions from these data. The mean values of AZ, CQ, and desethyl-CQ concentrations at 0 hours on Day 2 (prior to the third dose) and on Day 7 (after the third dose) that were observed in this paediatric study were similar to the concentrations at same PK sampling time points observed in African adults treated with 1,000 mg AZ plus 600 mg CQ once daily for three days. In the African study, the combination of AZ and CQ resulted in an overall efficacy rate of 100% at Day 28 [7].

The overall proportion of subjects with AEs was similar across treatment groups within each cohort, although treatment-related AEs were reported more frequently in the AZCQ group in both cohorts. Posthoc analysis of the frequency of AEs in cohort 2 showed that, for most, there was no statistically significant difference between treatment groups, however, vomiting was more frequent in the AZCQ group and abdominal pain was more frequent in the AL group. Study discontinuation due to AEs was higher in the AZCQ group in Cohort 2. No unexpected safety signals were identified beyond the known AE profile of individually dosed AZ or CQ.

In this study, efficacy of AZCQ was inferior to AL. Therefore, consideration of future use of AZCQ for IPT or seasonal malaria chemoprophylaxis may require additional study to determine whether a combination of AZ with other malaria drug(s) or use of an alternative dosing regimen with higher AZ dosing improves response to treatment with AZCQ in children aged six to 59 months.

## Additional file

Additional file 1:
**Median change from baseline to last observation* in laboratory values in cohort 2.**
