# Supplementary material for: Comparison of azithromycin plus chloroquine versus artemether-lumefantrine for the treatment of uncomplicated Plasmodium falciparum malaria in children in Africa: a randomized, open-label study
Source: Malar J. 2015 Mar 10;14:108. doi: 10.1186/s12936-015-0620-8 (PMC4358906; doi:10.1186/s12936-015-0620-8)
Supplement: Additional file 1: — Median change from baseline to last observation* in laboratory values in cohort 2. [file 12936_2015_620_MOESM1_ESM.docx]

**Additional file 1: Table S1 Median change from baseline to last observation* in laboratory values in cohort 2**

|  | **AZCQ** | | | **AL** | | |
| --- | --- | --- | --- | --- | --- | --- |
|  | **N** | **Baseline median** | **Median change from baseline** | **N** | **Baseline median** | **Median change from baseline** |
| Hemoglobin, g/dL | 122 | 13.2 | 1.1 | 128 | 13.6 | 0.8 |
| Hematocrit, % | 122 | 37.8 | -1.1 | 130 | 38.0 | -1.1 |
| RBC count 10^6^/mm^3^ | 122 | 4.76 | -0.12 | 130 | 4.73 | -.011 |
| Platelets, 10^3^/mm^3^ | 122 | 199 | 20 | 130 | 193 | 30 |
| WBC count, 10^3^/mm^3^ | 122 | 7.6 | 0.1 | 130 | 6.8 | 0.0 |
| Lymphocytes, absolute count, 10^3^/mm^3^ | 122 | 1.60 | 0.68 | 130 | 1.62 | 0.62 |
| Total neutrophils, absolute count, 10^3^/mm^3^ | 122 | 4.81 | -1.50 | 130 | 4.77 | -1.14 |
| Basophils, absolute count, 10^3^/mm^3^ | 33 | 0.06 | 0.01 | 37 | 0.05 | 0.02 |
| Eosinophils, absolute count, 10^3^/mm^3^ | 33 | 0.03 | 0.04 | 37 | 0.03 | 0.05 |
| Monocytes, absolute count, 10^3^/mm^3^ | 122 | 0.55 | 0.09 |  | 0.46 | 0.11 |
| Bilirubin Total, mg/dL | 122 | 1.0 | -0.4 | 130 | 0.8 | -0.3 |
| AST (SGOT), IU/L | 96 | 39 | -8 | 104 | 39 | -3 |
| ALT (SGPT), IU/L | 122 | 25 | -2 | 130 | 23 | -3 |
| Creatinine, mg/dL | 122 | 0.9 | 0.0 | 130 | 1.0 | -0.0 |

*Last observation is defined as last observation while on study drug, including up to 35 days after the last dose.

AL=artemether-lumefantrine; ALT = alanine aminotransferase; AST = aspartate aminotransferase; AZCQ; RBC= red blood cell; SGOT = serum glutamic oxaloacetic transaminase; SGPT = serum glutamic pyruvic transaminase; WBC = white blood cell*.*
